# Clinical and Biological Characterization of Skin Pigmentation Diversity and Its Consequences on UV Impact

**DOI:** 10.3390/ijms19092668

**Published:** 2018-09-08

**Authors:** Sandra Del Bino, Christine Duval, Françoise Bernerd

**Affiliations:** L’Oréal Research and Innovation, 1 avenue Eugène Schueller, 93601 Aulnay-sous-Bois, France; cduval@rd.loreal.com (C.D.); fbernerd@rd.loreal.com (F.B.)

**Keywords:** constitutive skin pigmentation, phototype, melanocyte, UV sensitivity, pigmentary disorders

## Abstract

Skin color diversity is the most variable and noticeable phenotypic trait in humans resulting from constitutive pigmentation variability. This paper will review the characterization of skin pigmentation diversity with a focus on the most recent data on the genetic basis of skin pigmentation, and the various methodologies for skin color assessment. Then, melanocyte activity and amount, type and distribution of melanins, which are the main drivers for skin pigmentation, are described. Paracrine regulators of melanocyte microenvironment are also discussed. Skin response to sun exposure is also highly dependent on color diversity. Thus, sensitivity to solar wavelengths is examined in terms of acute effects such as sunburn/erythema or induced-pigmentation but also long-term consequences such as skin cancers, photoageing and pigmentary disorders. More pronounced sun-sensitivity in lighter or darker skin types depending on the detrimental effects and involved wavelengths is reviewed.

## 1. Skin Pigmentation Diversity

### 1.1. Evolution of Skin Pigmentation Diversity

**Figure 1:**
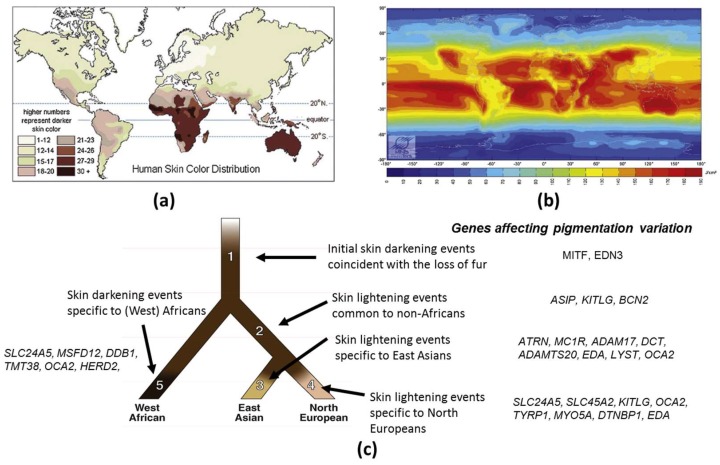
Skin color and UV distribution: (**a**) World map of skin color. Data for native populations collected by R. Biasutti prior to 1940 (http://anthro.palomar.edu/vary/vary_1.htm) show that darker skin types can be found mostly between 20° north and south of the equator; (**b**) World map of yearly mean of daily irradiation in UV (280–400 nm) on horizontal plane in J/cm^2^ averaged over the period (1990–2004) (http://www.soda-is.com/eng/map/maps_for_free.html) computed from satellite imagery, Mines ParisTech/Armines 2006; (**c**) Speculative evolutionary tree model for human skin pigmentation in three populations (adapted from [3]). Shading on the branches shows deduced pigmentation levels of populations. Genes hypothesized to have been subject to positive selection are listed.

Skin pigmentation is one of the most variable and noticeable phenotypes in humans, but the genetic basis and evolution of this polygenic trait has not yet been fully unraveled. The available evidence strongly suggests that the observed variation in this trait within and between populations has been shaped by natural selection. The general geographic patterns of skin pigmentation show a strong correlation with latitude and ultraviolet radiation (UVR) intensity. Skin pigmentation tends to be darker in equatorial and tropical regions (Sub-Saharan Africa, South Asia, Australia and Melanesia) where UVR levels are higher than in regions distant to the equator (Figure 1a,b). The current evolutionary hypothesis for skin pigmentation variation is the vitamin D/folate hypothesis, where a compromise may exist between the requirements for photoprotection on the one hand, and vitamin D3 synthesis on the other hand [1]. Dark skin would have a biological advantage under high UVR to protect from UV-induced sunburn, skin cancer, immune suppression and photolysis of folate, a metabolite essential for the development of embryonic neural tube and fertility, while light skin would have a biological advantage in regions far from the equator exposed to lower levels of UVR where UVB is needed and corresponds to the effective wavelengths for transformation of 7-dehydrocholesterol to vitamin D3 in the skin, and results in multiple effects on health, ranging from bone metabolism, innate immune response, cell proliferation and differentiation to fertility [2].

As described in the next section, skin color is determined mainly by epidermal melanin pigments, with a minor contribution from carotenoids and (de)oxyhemoglobin from dermal capillaries [4].

### 1.2. Melanocyte Biology

Melanin pigments are produced in the epidermis by highly specialized cells, the melanocytes, neural crest-derived cells migrating as melanoblasts, during embryogenesis, into the epidermis and hair follicles via the mesenchyme. Melanogenesis is a very complex process with multiple regulatory agents and pathways that could interact within the melanocyte but also in cooperation with the other cutaneous cells types (for a general review on melanogenesis, see [5]). Localized at the dermal–epidermal junction, each interfollicular melanocyte is connected through its dendrites to approximately 40 keratinocytes, establishing the so-called epidermal melanin unit [6]. Within melanocytes, melanin is synthesized in distinct, specialized membrane-bound organelles called melanosomes. Variations in constitutive pigmentation are attributed to differences in quality and quantity of the melanin content as well as in the size, amount, distribution and fate of melanosomes within the epidermal melanin unit rather than to differences in melanocyte density, as the latter is quite comparable among different ethnicities [7,8,9]. Melanocytes produce two chemically distinct types of melanin: the brown-black eumelanin, and the yellow-red pheomelanin [10,11,12,13,14]. The type of melanin produced depends on the function of melanogenic enzymes and the availability of substrates. Tyrosinase (TYR) is the key enzyme in melanogenesis and mediates the first steps in melanin synthesis, which involve the hydroxylation of the amino acid tyrosine to l-3,4-dihydroxyphenilalanine (DOPA) and subsequent oxidation to DOPAquinone. The latter is a key compound that undergoes further modification in two different pathways depending on the availability of the amino acid cysteine [15,16]. Cysteine reacts with DOPAquinone to produce 5-*S*-cysteinyldopa (5SCD) and 2-*S*-cysteinyldopa (2SCD). Cysteinyldopas are then oxidized by DOPAquinone to give benzothiazine intermediates, which gradually polymerize to form pheomelanin pigment. In the late stage of pheomelanin production, the benzothiazine moiety is gradually converted to benzothiazole moiety [17]. When cysteine is consumed in melanosomes, DOPAquinone spontaneously reacts to give, via DOPAchrome, 5,6-dihydroxyindole (DHI) and 5,6-dihydroxyindole-2-carboxylic acid (DHICA). The production of DHICA is accelerated by DOPAchrome tautomerase (DCT), also known as tyrosinase-related protein 2 (TRP2) or copper ions [18]. These 5,6-dihydroxyindoles are then further oxidized by tyrosinase and tyrosinase-related protein 1 (TRP1) to produce the eumelanin polymer.

It is generally accepted that eumelanin is photoprotective by limiting the extent of UV penetration within the epidermis and scavenging reactive oxygen radicals. In contrast, pheomelanin is not only weakly photoprotective against UV but also highly phototoxic and can enhance the UV-induced production of reactive oxygen species and further damage the cells [19,20,21,22].

Melanin synthesis takes place in melanosomes, a major advantage as it isolates melanogenesis from the rest of the cell and avoids the toxic effect of reactive quinoid melanin intermediates. There are four different maturation stages (I–IV) with increasing degree of melanization [23]. Rising from endoplasmic reticulum, Stage I melanosomes correspond to perinuclear and spherical premelanosomes where the internal matrix formed by the structural protein Pmel17 (gp100) begins to assemble. Stage II melanosomes are ovoid organelles containing longitudinally organized matrix fibers but without the presence of melanin. Stage III melanosomes show deposits of melanin along the matrix striations and tyrosinase activity is maximal. Stage IV melanosomes are opaque, filled with melanin and have no longer tyrosinase activity. 

Besides tyrosinase and related proteins, melanosomal pH is also important in the regulation of skin pigmentation. Melanosomes derived from light skinned melanocytes are more acidic and have lower tyrosinase activity than melanosomes from dark skin which have more neutral pH and higher tyrosinase activity [24,25]. Actually, the activity of tyrosinase is greatly enhanced at neutral pH. Melanosomal pH regulating tyrosinase activity is thus a key parameter in the melanogenesis process. In darker skin types, it has been shown that Tyrosinase activity is increased [26] but without up-regulation of *TYR* gene expression [9]. To date, several ion channels, pumps and transporters have been identified as effectors of melanosomal pH among them ATP7A, a copper transporting ATPase, V-ATPase, a H^+^ pump, and the more specific OCA2/p protein, a chloride anion channel, SLC45A2/MATP a sugar transporter-like membrane protein, SLC24A5-encoded NCKX5 protein, a putative NA^+^/Ca^2+^ion exchanger pump and the Two Pore Channel 2 TPC2 (*TPCN2* gene), a cation (Na^+^) ion channel [27]. 

More than 125 genes are known to regulate pigmentation. They regulate differentiation, survival of melanocytes, biogenesis and melanosome function which require a number of specific enzymes and structural proteins to mature and produce melanin. 

The critical enzymes include tyrosinase (TYR), TRP1 and DCT, mutations of which dramatically affect the quality and quantity of melanin produced. Critical structural proteins include Pmel17 and MART1 both required for maturation of melanosomes l-tyrosine and l-DOPA, besides serving as substrates and intermediates of melanogenesis, are described to be also bioregulatory agents acting as positive regulators of melanogenesis enzymes and as regulators of melanocyte functions (e.g., proliferation) [28].

Hormonal regulation of pigmentation is crucial and one of the major determinants of pigment phenotype of the skin is the melanocortin 1 receptor (MC1R), a G protein-coupled receptor that plays a key role in the switch between eumelanin and pheomelanin synthesis within the melanocytes [29,30]. MC1R function is controlled by the agonists α-melanocytes-stimulating-hormone (α-MSH) and adrenocorticotropic hormone (ACTH) and by an antagonist, Agouti signaling protein (ASIP) [5]. Binding of MC1R by an agonist activates adenylyl cyclase, and increases levels of intracellular cAMP that activates protein kinase A (PKA) which in turn leads to increased transcription of microphthalmia-associated transcription factor (MITF) and, as a consequence, increased transcription of tyrosinase. Ultimately, this leads to an increased tyrosinase activity, resulting in eumelanin synthesis. Conversely, when ASIP binds to MC1R, there is a decreased tyrosinase activity resulting in pheomelanin production. Of note, MITF acts as a master regulator of melanocyte development, function and survival [31] and plays a role in the control of melanogenesis that leads to increased transcription of a range of genes including *TYR*, *TRP1* and *TRP2* involved in the control of the type and amount of melanins produced. 

### 1.3. Genetic Basis of Skin Pigmentation Diversity

Constitutive skin pigmentation is a polygenic trait and, in recent years, the number of genes and the allelic variants that affect human skin pigmentation that have been identified has significantly increased. Studies of human pigmentation disorders, animal pigmentation models combined with the identification of regions in the human genome under positive selection in different populations, and genome-wide association studies (GWAS) have identified several single nucleotide polymorphisms (SNP) involved in human pigmentation, including SNPs that had not previously been implicated in pigmentation [32] (Table 1). 

Approximately 15 genes have been associated with skin pigmentation variation in humans and some of the major ones are described as follows.

The melanocortin-1 receptor gene (*MC1R*) is the most well studied. This short intronless gene plays a key role in the switch between eumelanin and pheomelanin synthesis within the melanocytes. *MC1R* is highly polymorphic in European populations with more than 60 variants having been described. Several *MC1R* variants are associated with fair skin and red hair, sun-sensitivity and melanoma risk in Europeans [43]. In East and Southeast Asian populations, some polymorphisms have been found. Conversely, *MC1R* polymorphisms are absent in sub-Saharan African populations and very low in other dark skinned populations such as Papuans or South Asians showing that *MC1R* is under strong functional constraint in regions of high UVR [44].

The solute carrier family 24 member 5 (*SLC24A5*) gene is a cation exchanger that has a key role in melanosome morphogenesis and melanogenesis. Variants in *SLC24A5* play a central role in skin lightening in Europeans, explaining approximately 30% of the difference in skin pigmentation between Europeans and West African populations [45]. The ancestral SNP (rs1426654 G) is present at very high frequencies in Sub-Saharan Africa, East Asia, Southeast Asia, the Americas, and Melanesia while the derived SNP (rs1426654 G>A) is found in Europeans and in geographically near populations in the Middle East, North Africa and Pakistan [35,45].

Another remarkably polymorphic gene is the membrane-associated transporter protein (*MATP*) also known as *SLC45A2*. In humans, mutations at *MATP* are responsible for oculocutaneous albinism type 4 (*OCA4*). *SLC45A2* shows a pattern of polymorphisms very similar to that of *SLC24A5*: the derived SNP (rs16891982 C>G) is found in European and closely related populations while the ancestral SNP (rs16891982 C) is associated with darker pigmentation [35].

The oculocutaneous albinism type 2 (*OCA2*) gene has been under positive selection in Europe and East Asia, but different alleles have been selected in each region. The derived allele *OCA2* rs1800404 C>T associated with light pigmentation occurs at high frequencies in European and East Asian populations whereas the ancestral allele (rs1800404 C) associated with dark pigmentation is highly frequent in Sub-Saharan Africans and Melanesians [35]. Furthermore, *OCA2* variant rs1800414 A>G is prevalent in most East Asian populations and has reached very high frequencies in Chinese, Japanese and Korean populations but is absent in African and European populations [39,46]. Another hypofunctional *OCA2* variant allele (rs74653330 G>A) was reported in East Asian populations living at highest latitudes (northern China and Mongolia) but was not observed in South Asian, European and African populations [47,48]. *OCA2* is a major gene conferring skin lightening among East Asians, indicating that the evolution of light skin occurred, at least in part independently, in East Asians and European populations [39,48].

The agouti signaling protein (ASIP) is the antagonist of MC1R and upon binding to this receptor, ASIP promotes the synthesis of pheomelanin. The *ASIP* ancestral allele (rs6058017 G) resulting in decreased antagonism of α-MSH binding to MC1R and thus increased eumelanin synthesis has been reported to be associated with dark hair and brown eyes in European-Americans [49] and was found also associated with dark skin in African Americans [41], while rs6058017 G>A polymorphism leads to lighter skin color. 

An interferon regulatory factor-4 (*IRF4*) SNP (rs12203592) is strongly associated with skin color, skin tanning response, freckling and nevus count [34,50,51,52]. The rs12203592 C>T polymorphism leads to reduced *IRF4* gene expression and consequently reduced *TYR* expression, leading to sun sensitivity and blue eyes [53]. While Chinese, Japanese and African populations are homozygous for the rs12203592 C allele, only European populations harbor rs12203592 C>T polymorphisms [42]. This SNP shows a north–south gradient allele frequency distribution across Europe [54]. 

The tyrosinase gene (*TYR*) encodes a key enzyme in melanogenesis and is responsible for oculocutaneous albinism type 1 (OCA1). It is also involved in normal pigmentation variation. Two polymorphisms (rs1042602 C>A and rs1126809 G>A) associated with reduced tyrosinase enzyme activity appear at high frequency in Europeans and are absent in African populations [33]. 

In East Asian populations, ADAM metallopeptidase domain (*ADAM17*) SNP (rs4328603 C>T), ADAM metallopeptidase with Thrombospondin type 1 motif 20 (*ADAMTS20)* SNP (rs11182091 C>T, rs11182085 A>G, rs1510523 C>T) and attractin (*ATRN*) show strong specific signatures comparable to the *SLC24A5* and *SLC45A2* signatures in European populations [39]. Additional examples of East Asian-specific pigmentation-associated alleles include *TRP1* (rs10809814) and *DCT* (rs1407995) [32,55].

Nevertheless, despite distinct genes and derived alleles in Europeans and East Asians, some genes, i.e., KIT Ligand (*KITLG*), *ASIP* and basonuclein (*BNC2*), have variants highly frequent in both populations.

Based on clear evidence for the selection of pigmentation-related genes in different populations, an evolutionary tree model for human skin pigmentation has been proposed in three populations East Asian, European and West African [3] (Figure 1c). This model suggests that skin lightened independently in Europeans and East Asian populations. The human tree starts with a hominin ancestor in Africa who probably had lightly pigmented skin covered with dark black hair that protected his skin from UVR [56]. As the lineage leading to the early Homo sapiens began to lose body hair, a strong selection for skin darkening emerged [57,58] (Branch 1). Microphtalmia-associated transcription factor (*MITF*) and endothelin 3 (*EDN3*) are the genes hypothesized to have been subject to positive selection. Later, the African and the European/East Asian populations diverged upon the “out of Africa” expansion (Branch 2), followed by the splitting of the East Asians and Europeans (Branches 3 and 4, respectively). The genetic mechanisms resulting in skin lightening in both Europeans and East Asians occurred independently, and after their divergence [35,45]. In Europeans, genetic selection for the evolution of light skin color has been confirmed for *SLC24A5* [35,45] and *SLC45A2* (*MATP*) [35,59]. In addition, myosin VA (*MYO5A*), dystrobrevin binding protein 1 (DTNBP1), *TYRP1*, ectodysplasin A (*EDA*), *OCA2* and *KITLG* may have undergone European-specific changes and may thus have also affected skin lightening in Europeans (Branch 4). A parallel mechanism driving skin lightening (convergent evolution) may have occurred in East Asians (Branch 3). In particular, *ADAM17* and *ADAMTS20* show strong selection signatures in East Asian populations, such as *SLC24A5* and *MATP* in European populations. *DCT*, *MC1R*, lysosomal trafficking regulator (*LYST*), *EDA*, *OCA2* and *ATRN* also show relatively strong signatures of distinct East Asian selective events (Branch 3). However, it seems that gene selection was not that straight forward. Two additional genes, *ASIP* and *BNC2*, show evidence of selection in both Europeans and East Asians. This would suggest that these genes were selected prior to the divergence of these populations (Branch 2). Two other genes, *LYST* and *KITLG*, appear to have had selective events that may have started on Branch 2 and then continued on to Branches 3 and 4. West African populations also experienced adaptive pressure for skin darkening relative to the ancestral human population, but the authors found less evidence for this and have placed no genes on Branch 5. 

Recently, two studies have shed light on the architecture of skin pigmentation in African skin and on regions of the genome not previously associated with skin color [37,38]. Four regions of the human genome associated with skin color in African populations living in Ethiopia, Tanzania and Botswana have been identified in or near *SLC24A5*, *MFSD12* (Major facilitator superfamily domain containing 12), *DDB1* (Damage specific DNA binding protein 1)/*TMEM38* (Transmembrane protein 38) and *OCA2*/ *HERC2* (HECT And RLD domain containing E3 ubiquitin protein ligase 2) [37]. The SNPs with the strongest association with skin color in Africans was *SLC24A5*. The light variant allele (rs1426624A) already known to frequently occur in Europeans, Pakistani, Central Asians and North Indians [36] was shown to be common (28–50%) in Ethiopians and Tanzanians with Afroasiatic ancestry and at moderate frequency (5–10%) in South African and Botswanan populations with low levels of East Asian ancestry and recent European admixtures. This light pigmentation variant at *SLC24A5* was thus likely introduced into East Africa by gene inflow from non-Africans. The second strongest association with skin pigmentation is due to the *MFSD12* gene, a transmembrane solute transporter that locates to late endosomes and/or lysosomes in melanocytes and not to eumelanosomes. Depletion of MFSD12 increases eumelanin content. A third genomic region associated with pigmentation contains the *DDB1*/*TMEM38* genes. *DDB1* plays a role in DNA repair. The fourth region of significantly associated SNPs encompasses the *OCA2*/*HERC2* loci. Variants associated with dark pigmentation at *MFSD2*, *DDB1*, *OCA2* and *HERC2* are found in Africans, which are also shared by southern Asian and Australo-Melanesian populations. In contrast to the lack of variation at *MC1R* in Africans [60], both light and dark alleles at *MFSD2*, *DDB1*, *OCA2* and *HERC2* arose in hominid and continued to evolve. In southern African populations with considerably lighter skin than equatorial Africans, novel variants associated with skin pigmentation have been identified in canonical *SLC24A5*, *TYRP1* and non-canonical pigmentation genes, including near *SMARCA2* (SWI/SNF related, matrix associated, actin dependent regulator of chromatin, subfamily A, member 2)/*VLDLR* (Very low density lipoprotein receptor) and *SNX13* (Sorting nexin 13) [38]. *VLDLR* and *SMARCA2* have been implicated in pigmentation in model organisms and in vitro studies. *SNSX13* regulates lysosomal degradation and G protein signaling but has not previously been associated with skin pigmentation. Thus, although genetic sequencing has shed light on many but probably not all the genes, more variants involved in variation of skin pigmentation in human populations still remain to be discovered.

### 1.4. Clinical Classification of Skin Pigmentation

There is a need for an objective tool to classify skin pigmentation in dermatological and cosmetic research. Furthermore, an accurate description of the color phenotype is necessary to compare different genetic studies of human skin pigmentation or to determine the role of skin color in responses to UVR. 

#### 1.4.1. Fitzpatrick Classification

**Figure 2:**
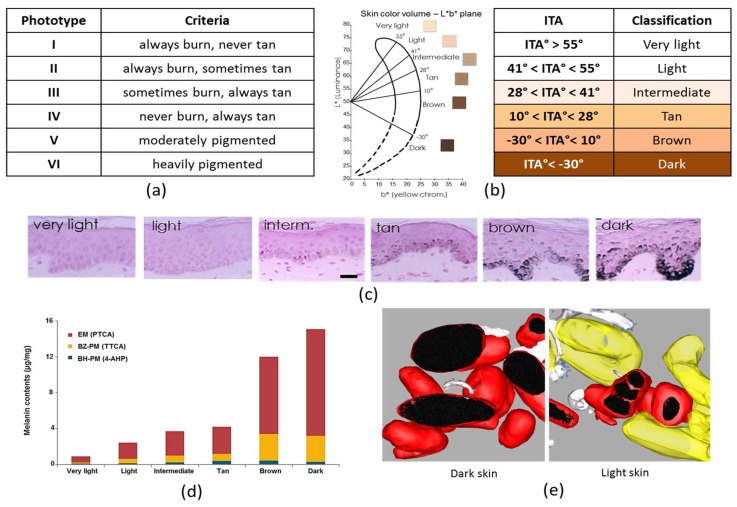
Classification of skin pigmentation: (**a**) Fitzpatrick classification; and (**b**) Individual typology angle (ITA)-based classification; (**c**) Melanin content in Fontana–Masson stained skin sections classified according to their ITA. scale bar = 50 µm; (**d**) Eumelanin (PTCA) and pheomelanin (TTCA and 4-AHP) content in each skin color group defined by ITA (adapted from [78]); (**e**) 3D models of isolated melanosomes in dark skin and clusters in light skin (adapted from [79]).

Skin color is usually defined by the Fitzpatrick’s phototype classification created in 1975 [61] (Figure 2a). This classification is based on a self-reported questionnaire, in which individuals grade their erythema sensitivity and tanning ability, respectively, 24 h and seven days after the first unprotected sun-exposure in the early summer. It was originally created to categorize Caucasian skin into four skin phototypes (SPT), I–IV, with decreasing erythema sensitivity and increasing tanning ability. Skin type V was added afterwards for individuals with brown skin of Asian and Latin American origin, and skin type VI for dark skin of African extraction. Skin types I–IV are thus based on clinical response to UV, whereas classification into types V–VI is based on constitutive pigmentation or ethnic origin. Although this classification is helpful to determine the appropriate dose for phototherapy and is widely used in epidemiological studies of skin cancers and in experimental and clinical photobiology, it is limited by its subjective and qualitative nature. Self-reporting is prone to recall errors; evidence shows that individuals typically underestimate their UV skin sensitivity [62] and on repeat questioning a few months later, only two out of three self-classified in the same phototype [63]. The classification is also based on ethnic origin and is irrelevant for all skin types, especially non-Caucasian, i.e., African [64,65], Asian [66,67,68], Arab/Middle East [69] or Hispanic skin types [65] as well as multiethnic populations. Furthermore, due to unprecedented mixing of ethnic populations today, ethnic origin is no longer a representative criterion for classifying human populations. 

Other more objective systems have been created to measure constitutive pigmentation. These include spectrometry and colorimetry [62,70,71] or skin color identification charts [72,73].

Currently, the main skin chromophores (melanin and hemoglobin) can be studied by three strategies based on reflectance technology: tristimulus colorimetry, specialized narrow-band reflectometry, and diffuse reflectance spectroscopy. 

#### 1.4.2. Individual Typology Angle-Based Skin Color Type Classification

An alternative skin color type classification tool was developed using the Individual Typology Angle (ITA) [74]. This classification is based on colorimetric parameters of the Commision Internationale de l’Eclairage (CIE) L*a*b* system established in 1976 to correlate to the vision of the human eye. In this system, any color can be represented by three variables: L* the lightness axis, a* the red-green axis and b* the yellow-blue axis, which can be plotted in three-dimensional space [75]. Tristimulus colorimeters such as Minolta Chroma Meter series (Konica Minolta Sensing, Osaka, Japan) or Check (Datacolor, Montreuil, France) are designed to approximate human vision and can be used to measure L*a*b* parameters. Erythema (redness of the skin primarily due to hemoglobin) is typically evaluated using the a* parameter.To measure pigmentation the L* parameter that varies from black (value 0) to white (level 100) and the b* parameter that ranges from yellow (positive value) to blue (negative value) are used to calculate the Individual Typology Angle (ITA) according to the formula: °ITA = [arctan(L*−50)/b*] × 180/3.14159. Using this formula, skin color types can be classified into six groups, ranging from very light to dark skin: very light > 55° > light > 41° > intermediate > 28° > tan > 10° > brown > 30° > dark [71] (Figure 2b). This classification is an objective and quantitative measure of skin color, irrespective of ethnic origin. Furthermore, it is physiologically relevant when applied to individuals of different geographic areas [76,77]. The ITA colorimetric classification showed that Caucasian skin types in France, USA and Russia are Light, Intermediate and Tan. African skin types in France and USA, classified SPT VI, are Intermediate to Dark. Hispanic skin types in Brazil, Mexico and the USA, classified SPT V, are heterogenous, ranging from Light to Brown. ITA values of Asian skin types, classified SPT V by Fitzpatrick, showed that in Northern East Asian countries, Japan and China, skin types are Light and Intermediate while in Southern East Asian countries, India and Thailand, skin types showed a wide variety in the degree of pigmentation ranging from Light to Dark skin groups. The results illustrate the differences that exist between a precise evaluation of the constitutive pigmentation using colorimetric values and the use of Fitzpatrick’s classification system, originally developed for Caucasian skin, and with limited validity for darker skins.

#### 1.4.3. Specialized Narrow-Band Reflectometry

Specialized narrow-band reflectometry has been developed to measure hemoglobin and melanin in the skin [80]. Hemoglobin has absorption peaks in the green-yellow region of the spectrum and absorbs very little in the red wavelengths, while melanin absorption decreases linearly in the red range [81]. The red reflectance yields an estimate of the melanin content and allows the calculation of the melanin index (MI). Subtracting the red absorbance due to melanin from the green reflectance estimates the erythema and gives the erythema index (EI). Commercially available narrow-band spectrometers include the DermaSpectrometer (Cortex Technology, Hadsund, Denmark) or the Mexameter (Courage-Khazaka Electronic, Köln, Germany). 

#### 1.4.4. Diffuse Reflectance Spectroscopy

With diffuse reflectance spectroscopy (DRS) reflectance values are measured at high optical resolution through the full visible spectrum which allows to separate the effects of melanin and hemoglobin because of their different spectral properties: melanin can be estimated by DRS from the slope of absorbance at 620–720 nm and hemoglobin from the absorbance at 560–580 nm [82]. DRS can also calculate parameters in the CIELab color space or other standard color spaces.

In genetic studies of human skin pigmentation, skin color variation is mostly assessed using DermaSpectrometer [35,36,37,38,39,83,84,85,86] or tristimulus colorimeter [40,87,88,89] rather than Fitzpatrick classification [34,43,53,90,91]. Interestingly, comparative studies of skin pigmentation using tristimulus colorimeters and narrow-band spectrophotometers, showed good correlations between L* and MI and between ITA and MI [92,93]. 

### 1.5. Analysis of Melanins and Melanosomes in Skins of Variable Pigmentation

Measurements of melanin in human skin and particularly in skin with different constitutive pigmentation are scarce [94,95,96,97,98], as compared to melanin analysis in mice, cell culture or hair [12]. 

#### 1.5.1. Melanin Content

Total melanin content in the epidermis can be quantified on Fontana–Masson stained skin sections. Total melanin content of skin samples with variable constitutive pigmentation was quantified [78] (Figure 2c). The melanin index (MI) was estimated in both the whole epidermis and the basal layer where it is produced by melanocytes, as the surface covered by the melanin staining in the epidermis including the stratum corneum or in the basal layer, over the surface of the epidermis or of the basal layer, respectively. Results showed a good correlation between MI in the whole epidermis or more specifically in the basal layer and ITA-based constitutive pigmentation (*R*^2^ = 0.87, *p* < 0.0001 and *R*^2^ = 0.89, *p* < 0.0001 respectively).

Total melanin content can also be estimated indirectly by spectrophotometry, after dissolving epidermal samples with Soluene-350 and measuring the A500 absorbance value [99]. The same samples with variable constitutive pigmentation were analyzed and the results showed that values of total melanin level highly correlated with the ITA value (*R*^2^ = 0.83, *p* < 0.0001) [78].

#### 1.5.2. Melanin Type

To discriminate pigment types, chemical degradation or solubilization of pigment is required. Eumelanin and pheomelanin content can be estimated by high-performance liquid chromatography (HPLC) after hydrogen peroxide (H_2_O_2_) oxidation and hydroiodic acid (HI) hydrolysis [100,101,102]. Eumelanin content can be assessed by measuring the content of pyrrole-2,3,5-tricarboxylicacid (PTCA), degradation product of DHICA melanin, while pheomelanin can be estimated by measuring 4-amino-3-hydroxyphenylalanine (4-AHP), degradation product of benzothiazine-type pheomelanin [101,102]. One study demonstrated lower concentrations of eumelanin (estimated as PTCA) in SPT I and higher levels in SPT II and III while there is no relationship to skin type for pheomelanin (4-AHP) [97]. Another study found the lowest concentrations of eumelanin (PTCA) in SPT I and II, the highest levels in SPT III and IV and the lowest concentrations of pheomelanin (4-AHP) in SPT I and II [98]. Another group described a good correlation between eumelanin (PTCA) and melanin content determined by Fontana Masson staining in skin specimens from subjects of different ethnic groups and SPT I–VI but no correlation with pheomelanin (4-AHP) [96]. Higher eumelanin (PTCA) and pheomelanin (4-AHP) content in human epidermis from deeply pigmented non-Europeans compared to lightly pigmented Europeans was also described [95]. Both eumelanin (PTCA) and pheomelanin (4-AHP) were found correlated with skin color measured as L* in skin types with a majority of northern European origin and four of Asian origin [94]. 

4-AHP has been used as a specific marker for pheomelanin, especially benzothiazine-type but thanks to the improvement of the chemical methodologies, thiazole-2,4,5-tricarboxylic acid (TTCA) can also be measured and is a good marker for benzothiazole-type pheomelanin [17]. It has recently also been taken into account for the quantification of pheomelanin in a rather large number of skin samples with a broad distribution of pigmentation intensities [78]. Analysis showed a good correlation between ITA and PTCA (*R*^2^ = 0.85, *p* < 0.0001) and for pheomelanin a good correlation between ITA and benzothiazole-type (TTCA) (*R*^2^ = 0.72, *p* < 0.0001) but a poor correlation between ITA and benzothiazine-type (4-AHP) (*R*^2^ = 0.24, *p* < 0.0001) (Figure 2d), confirming and extending earlier findings described above. A good correlation between total melanin, eumelanin and pheomelanin combined, and ITA (*R*^2^ = 0.84, *p* < 0.0001) was also showed by HPLC.

Although, until recently, it was considered that eumelanin accounts for more than 90% of total melanin [94], this study that incorporated the contribution from benzothiazole-type pheomelanin in the estimation of pheomelanin level revealed for the first time that the human epidermis comprises approximately 74% eumelanin and 26% pheomelanin, regardless of the degree of pigmentation, the pheomelanin being mostly of the benzothiazole type. 

Melanin can also be detected by electron paramagnetic resonance (EPR) spectrometry, due to stable free semiquinines-type radicals within its structure. EPR is similar to nuclear magnetic resonance in which electron spins are detected instead of nuclear spins. Due to slightly different EPR signals of eumelanin and pheomelanin, this method has been suggested to enable a distinction between both types of melanins in skin or hair samples and pigmented malignant melanomas (for more details see [103]). However, due to technical limitations this method is not applicable for ex vivo samples and is mostly used for research in the field of melanoma. No precise characterization of the different EPR signals could be found in skin of different color types. 

#### 1.5.3. Melanosome Transfer, Distribution and Organization in Skins of Variable Pigmentation

Once matured, melanosomes are transferred from melanocytes to keratinocytes through the dendrites. Four main mechanisms involved in this complex process have been proposed and may coexist: (1) cytophagocytosis of the dendrite tip by the keratinocyte; (2) fusion of the melanocyte and keratinocyte plasma membrane; (3) shedding of the plasma membrane-enclosed melanosome-rich packages followed by phagocytosis; and (4) exocytosis/endocytosis of melanocore [104]. Most widely described, phagocytosis can be promoted by two keratinocyte cell surface receptors, Proteinase activated receptor (PAR-2) and Keratinocyte growth factor receptor (KGFR), differentially expressed in skin of different colors. Keratinocytes from dark skin displayed higher level of PAR-2 expression and activation compared to light skin [105]. Inversely, a higher KGFR expression in light keratinocytes compared to dark keratinocytes was described unveiling the more pronounced effectiveness of KGF on the stimulation of melanosome transfer in light keratinocyte under UVB stimulation [106]. 

Within keratinocytes, the quantity, distribution pattern and fate of melanosomes vary among ethnic groups. Early electronic microscopic studies described isolated melanosomes in basal keratinocyte from dark skin whereas, in light skin, they appeared clustered together in membrane-limited groups of two to eight melanosomes [107,108]. Melanosomes, isolated or in clusters, are found predominantly over the nucleus of keratinocytes where they form a microparasol which provides photoprotection against sun radiation-induced DNA damage. These observations were confirmed in dark (SPT IV African/American) skin and in light (SPT II Caucasian) skin and demonstrated that melanosomes within keratinocytes of Asian (SPT IV–V Chinese) skin are distributed individually and in clusters [109]. Melanosome size varies with ethnicity, African skin has the largest melanosomes, European skin the smallest, and the melanosomes in Indian, Mexican and Chinese skin being intermediate in size [10]. In a recent study [79], melanosomes were systematically characterized on a skin panel representative of skin color diversity and objectively classified according to the ITA value. The results quantitatively confirmed at the melanosome level, the pigmentation gradient in the overall epidermis, from the basal layer to the stratum corneum, between skin types but also showed that whatever the skin phenotype, keratinocytes in the basal layer contain most melanosomes (60–80% of the total melanosomes). In addition, using high resolution electronic microscopy and tomographic reconstruction, the melanosome pattern was clarified by revealing that in basal layer only, clusters of small melanocores (melanosome deprived of melanosomal membrane) and single large melanocores, both surrounded by an outer single membrane, coexist in the different skin types. However, the ratio between cluster and single forms gradually decreases as skin pigmentation increases resulting in predominant melanocore clusters in light skin and single melanocores in dark skin (Figure 2e). It is worth mentioning that these data together with the work of Tarafder et al. [110] and Correia et al. [111] supported the exocytosis-endocytosis model of melanosome uptake. This distribution, single or in cluster, could be dictated by the origin of keratinocytes since when dark keratinocytes were cultured with light melanocytes, melanosomes were predominantly isolated whereas when light keratinocytes were put in contact with dark melanocytes, clusters of melanosomes increased [112,113]. Regarding the fate of melanosomes in the epidermis, the first hypothesis was that transferred melanosomes are degraded, particularly in light skin, as keratinocytes undergo differentiation, leading to the decrease or disappearance of melanosomes in the suprabasal layers [107,109,114,115]. This idea was supported by the fact that, in vitro, keratinocytes from light skin displayed accelerated melanosome loss compared to those from dark skin [114]. This potential degradation efficacy was attributed to the higher expression of hydrolytic enzymes in particular Cathepsin L2 in light epidermis [116,117], and to a higher autophagic activity of light compared to dark keratinocytes [115]. However, these results have been challenged with the recent findings of Correia et al. [111] and Hurbain et al. [79] who did not find autophagic or phagolysosomal markers associated with these organelles but conversely highlighted that these non-acidic melanocore clusters have contact with mitochondria and endoplasmic reticulum which may contribute to their homeostasis. A new paradigm is therefore raised of melanosome clusters being non-degradative protective organelles ensuring integrity and function of small melanocores. 

### 1.6. Other Biological Factors that Influence Skin Constitutive Pigmentation

Apart from melanogenesis regulation within melanocyte, much evidence is now available on the regulation of melanogenesis through the interactions and cross talk between melanocytes and other cutaneous cell types, mainly keratinocytes and dermal fibroblasts [118,119].

Paracrine regulation is now well established. One of the major mechanism of pigmentation regulation acts through the POMC local production and processing to CHR-related peptides, α-MSH, ACTH and β-endorphin [120]. Many other keratinocytes derived soluble factors, growth factors and cytokines have been identified such as basicFGF (bFGF), endothelin 1, IL1α, SCF, GM-CSF, HGF, GROα, PGE2/PGF2a and LIF (for review see [121]). Most of them have been clearly associated with melanocyte or melanogenesis activation upon UV exposure and some have also been suggested to play a role in the development of hyperpigmentary disorders, such as lentigo senilis [122]. On the other hand, some of them, such as cytokines such as IL-1, are also regulators of POMC expression [120]. However, few data have identified specific keratinocyte actors or derived factors associated with the level of constitutive pigmentation. A study from Yin et al. analyzed gene expression in the epidermis obtained from skins of African, Asian and Caucasian subjects. After separating the epidermis using the suction blister technique, differences between Asian and Caucasian skin could not be highlighted except for the microtubule associated gene *NINL* which was upregulated in Asian skin [123]. In contrast, a differential expression profile between Asian/Caucasian and African skin was found. Among the genes differentially expressed, some were already known to be associated with skin color type such as *FRZB*, *CDH12* or *KITLG*. Many of the modulated genes encode receptors and their ligands. This emphasizes the interactions between epidermal cell types, but at that stage no functional validation has been provided. Among the candidates, *CCL18* or *S100A4* showed a weaker expression in dark skin phototypes. The expression of protease-activated receptor-2 (*PAR-2*), has also been shown to be higher in darker skin types compared to fair skin phototypes, suggesting a role for melanosome phagocytosis in keratinocytes [105,124]. The role of transfer of melanosome in keratinocytes has also been highlighted by the higher expression of Rab27 in darker skin types [125].

The dermal compartment has also been shown to play a role in pigmentation regulation, through the extracellular matrix proteins such as laminins and collagen IV but also fibroblastic soluble factors. Some of these are common with keratinocytes i.e., SCF and HGF but others are specifically produced by dermal fibroblasts and can enhance melanin synthesis and melanocyte proliferation such as KGF/FGF7. The contribution of dermal fibroblasts to skin pigmentation has been clearly shown in three dimensional reconstructed skin models performed under various fibroblast conditions [126]. Dermal factors have also been identified to be abnormally expressed in pigmentary lesions, such as KGF/FGF7, HGF and SCF in lentigo senilis [127,128] and an increased secretion of SCF and HGF by dermal fibroblasts in neurofibromatosis type 1 (NF-1) disease [129]. Dermal soluble factors have also been demonstrated to contribute to constitutive pigmentation. Dermal fibroblasts from the lighter anatomical skin sites of the body, the soles and palms, were shown to produce higher levels of Dickkopf 1 (DKK1), a Wnt pathway antagonist compared to fibroblasts from other parts of the body [130,131] and a role of DKK1 in the down-regulation of melanocyte proliferation and melanin synthesis was demonstrated. The group of V. Hearing also addressed the question of the role of dermal fibroblasts in the level of constitutive pigmentation. They performed a cDNA array gene expression analysis on dermal fibroblasts isolated from skin of phototypes SPT I, III and VI. They were unable to find any difference in the expression of well-known fibroblastic regulators of melanogenesis such as SCF, bFGF or DKK1 but identified among the differentially expressed genes, Neuregulin 1 as a positive regulator for melanogenesis to be highly expressed in fibroblasts from skin type VI compared to those of lighter skin phototypes [132,133]. Among the mechanisms of cell–cell interactions, there is a growing interest in the role of secreted vesicles called extracellular vesicles (EVs), including the exosomes, the endosome-derived vesicles. Exosomes are secreted in the intercellular space and contain proteins, lipids and RNAs among them miRNAs, the small non coding RNAs known to regulate major biological functions [134,135] (for miRNAs, see [136]). Epidermal keratinocytes have been shown to release exosomes and their content differs depending on the stage of keratinocyte differentiation. Keratinocyte-derived exosomes may also be responsible for the dialog with dermal fibroblasts [134,135,136]. More recently, a characterization of EVs from keratinocytes from Caucasian and African skin types has been reported [137]. Apart from the classical exosomal component (histocompatibility complex class I, CD9, CD81, and Hsc70) and specific keratinocyte proteins such as keratins and stratifin, the authors analyzed the miRNAs profile. Although the amount of released exosomes did not differ between skin of different ethnic origin, exosomes from African skin keratinocytes play a role in the stimulation of pigmentation upon UV exposure but more interestingly, exert a melanogenesis stimulation on Caucasian melanocytes. This effect was associated with an increase in Tyr activity and MITF expression suggesting a role of Black keratinocytes in the regulation of pigmentation in Black skin. Among the components of these exosomes 30 miRNAs were differentially expressed between both ethnic skin origins among them miR203, the major miRNA already known to be implicated in keratinocyte differentiation [138] which showed a higher expression in exosomes from Black skin. MiR 203 has also been demonstrated to regulate pigmentation in melanoma cells but also in normal melanocytes by targeting Tyr and Kif5b without effect on MITF [139]. Other miRNAs have been shown to interfere with pigmentation and melanogenesis such as miR3196 or miR145 involved in the UVB response [137,140]. In patients with melasma, the down-regulation of miR675 stimulates pigmentation. MiR675 has been shown to be secreted in keratinocytes derived exosomes and has MITF as a direct target [141].

Although an increasing number of studies revealed the role of some miRNAs in the regulation of pigmentation and pigmentary disorders, the number of identified miRNAs involved in the regulation of constitutive skin color is still quite low. Some data have reported their functional role in animal coat color such as miR137 in mice [142] or miR25 in the skin of alpacas [143]. 

## 2. Acute Consequences of UV Exposure

UVR exposure causes acute consequences on the skin such as erythema and skin pigmentation as well as chronic consequences such as skin cancer, photoaging and pigmentary disorders. These effects are often highly dependent on skin color type and are reviewed in the next sections.

Considering the beneficial effect of sun exposure, i.e., vitamin D synthesis, it is now established that, in addition to the classical pathway of synthesis resulting from the conversion of 7-dehydrocholesterol to previtamin D in the skin by absorption of UVB photons (290–315 nm) [144], an alternative pathway of vitamin D activation occurs via CYP11A1 expression and may contribute to beneficial anti-proliferative and anti-inflammatory effects [145]. Considering the differences between skin color types, a systematic review of the role of melanin on vitamin D3 synthesis showed that, among 12 studies, seven reported less vitamin D3 in darker skins, and five found no difference in different skin types [146]. Although the authors concluded that pigmented skin seems to be less effective than fair skin in the synthesis of vitamin D3, this question requests more data to be clarified. 

### 2.1. Erythemal Reaction/Sunburn

Erythema or sunburn is a skin reddening caused by increased blood flow. It is the most widely used clinical endpoint in photobiology and is used as an indicator of UVR sensitivity by estimating the minimal erythemal dose (MED) as the lowest UVR dose able to induce a just perceptible skin redness (or redness with defined borders) 24 h post-exposure to a series of increasing UV doses [147]. 

Several studies have shown a relationship between MED and skin color, i.e., increased MED with skin type or skin color [96,148,149,150,151,152,153,154,155,156]. A powerful association between MED and skin colorimetric parameters and particularly the L* parameter was shown [157].

Dark skin has been shown to have an intrinsic sun protection factor (SPF) of 13.4, compared to 3.4 for light skin [158,159]. Additionally, dark skin transmits 7.4% of UVB compared to 29.4% in light skin [160]. These studies show that approximately four times as much UV reach the upper dermis of light skin compared to dark skin.

The relationship between various measures of constitutive skin pigmentation and erythema caused by solar-simulated radiation (SSR) UV, 290 and 310 nm UV was analyzed and a significant relationship (*p* < 0.001) between MED and skin color (SPT I-IV) was found using SSR and 310 nm UVB but not for 290 nm, thus suggesting that basal layer melanin only protects against erythema with wavelengths >290 nm [161].

**Figure 3:**
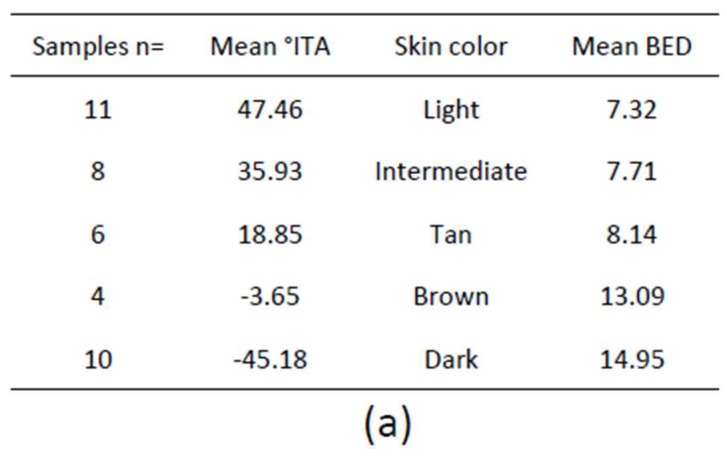

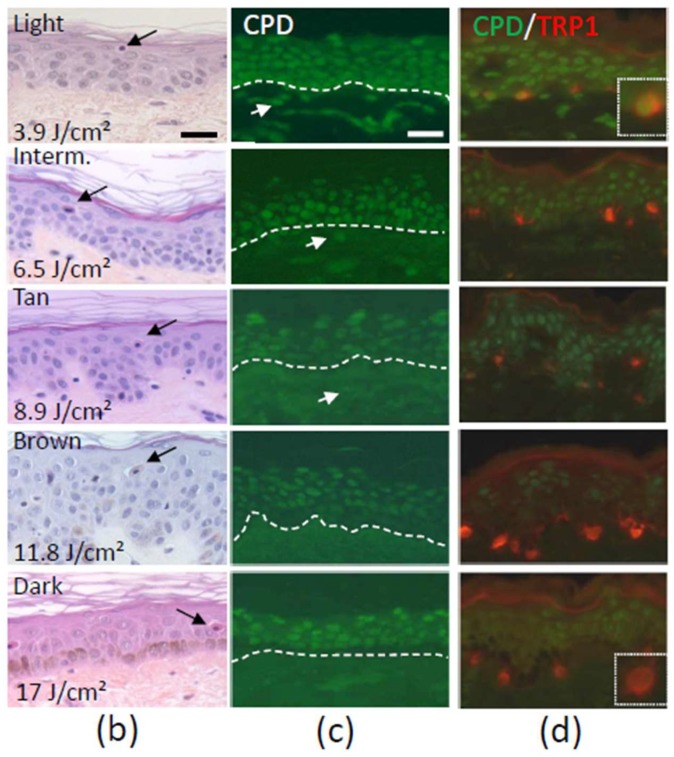
(**a**) Number, mean individual typology angle (ITA) value, skin color and mean biologically efficient dose (BED) of 39 skin samples; (**b**) Hematoxylin, eosin, saffron staining at the BED 24 h after UVR exposure. Typical sunburn cells (arrows) are shown; (**c**) Cyclobutane pyrimidine dimer (CPD) immunostaining at the BED immediately after UVR exposure. Nuclear accumulation of DNA damage in all the epidermal layers and the dermis is shown (arrows) for Light, Intermediate (Interm.) and Tan skin. Lesions are found in the suprabasal epidermal layers for Brown and Dark skin. Dotted lines delimitate the dermal-epidermal junction; (**d**) CPD (green) and tyrosinase-related protein (TRP1) (red) double detection at the BED show CPD-positive melanocytes in Light, Intermediate and Tan skin and a majority of CPD negative melanocytes in Brown and Dark skin. Dotted frames are magnifications of melanocytes. Bar = 25 µm.

Erythema is clinically associated with the induction of sunburn cells (SBC), i.e., apoptotic keratinocytes histologically characterized as dyskeratotic and vacuolated keratinocytes 24 h after UVR exposure [162]. SBC can be used as endpoints for photoprotection studies and are the basis of the biologically efficient dose (BED) which is the minimal UVR dose required to induce one SBC per 0.45 µm of the epidermis [163]. BED in vitro is equivalent to MED in vivo because SBC become apparent at 1 MED. SBC were quantified in skin samples of variable constitutive pigmentation 24 h after exposure to increasing SSR doses and the results showed a statistically significant correlation between skin color measured by ITA and BED (*R*^2^ = 0.70; *p* < 0001), i.e., the darker the skin the higher the BED (Figure 3a,b), similar to minimal erythemal dose MED [71].

### 2.2. DNA Damage

Cyclobutane pyrimidine (CPD) dimers are the major UV-induced DNA lesions in human skin and are indisputably the causal events in the development of skin cancers [164,165,166,167]. Several studies have demonstrated that UVR exposure induces DNA damage in all skin types and that the extent of such damage is inversely related to constitutive pigmentation of the skin [96,168,169,170]. CPD may trigger erythema, immunosuppression and photoaging that may increase the risk of skin cancer [171]. The action spectra for DNA damage (CPD), melanogenesis and erythema are very similar, suggesting that DNA is the chromophore for melanogenesis and erythema [147,172] and erythema can be considered as a clinical surrogate for DNA damage.

Skin color has been shown to influence the amount and distribution of DNA damage in the epidermis. An inverse relationship between melanin content and DNA damage induced by exposure to 1 MED of UVA/UVB was reported in individuals of different ethnic origin [96]. DNA damage-inducing effectiveness of a fixed sub-erythemal dose (100 J/m^2^) of UVA/UVB was compared on three ethnic groups Caucasian, Asian and African-American and showed a uniform distribution of CPD throughout the epidermis in lighter skin, while CPD declined with increasing distance from the skin surface in dark skin [168]. Dark skin provided a protection factor of approximately 3 for CPD. The DNA damage in the epidermis of different ethnic groups exposed to 1 MED of UVA/UVB was investigated and showed that CPD damage was similar in the upper and lower epidermis of Caucasian skin, while in the lower epidermis it was 1.5-fold and 2.1-fold lower than in the upper epidermis of Asian/Hispanic and African-American skin respectively, meaning that the upper epidermis of dark skin is more photoprotective than that of fair skin [173]. The erythema and CPD protection factor (PF) was determined by exposure to 2 MED SSR one week after stimulating tanning in skin type II and IV with repeated sub-erythemal SSR UV doses for two weeks [174]. The results showed that for both skin types PF were equivalent to 2–3, supporting the hypothesis that DNA is the chromophore for erythema as suggested by Young et al. [147]. Recently one study [175] determined the PF against DNA damage of constitutive pigmentation by comparing the SSR-induced CPD in buttock skin of fair skinned (SPT I/II *n* = 6) and dark skinned (West Africans, SPT VI, *n* = 6) participants and showed that melanin in dark skin provides protection against CPD by 8-fold in the whole epidermis and by 59-, 16.5- and 5-fold in the basal, middle and upper epidermis respectively. Although based on a small sample size and on limited skin color types, the basal and middle layer DNA protection factor are aligned with the relative differences in BCC (×60) and SCC (×20) of light and dark skin [176] (see Skin cancers section).

An inverse relationship between constitutive pigmentation measured by ITA and CPD on samples collected immediately after SSR UVR exposure was shown [71] (Figure 3c). Whereas for Light, Intermediate and Tan skin, DNA lesions were detected in all epidermal layers including the basal layer and the upper dermis, they were present only in the suprabasal layers in Brown and Dark skin. Lesions at the origin of potential mutations in the basal proliferative layer of the epidermis may explain, from a biological standpoint, the higher susceptibility of light skin types to develop UVR-induced skin cancers. The results also show that more pigmented skin, namely Intermediate and Tan skin types, comprising notably people of Hispanic, African-American and Asian origins, are also subject to this type of damage. 

DNA damage occurring specifically within melanocytes was also investigated in skin with different ethnic origin or constitutive pigmentation. A 7.5-fold higher CPD levels in melanocytes of Caucasian skin, and 2.9-fold higher in Asian/Hispanic skin compared to African-American skin was shown one day after a single 1 MED UV dose [173]. A dose-dependent increase in DNA lesions within melanocytes of skins with variable constitutive pigmentation measured by ITA (Figure 3d) and prevalence of CPD-positive melanocytes in Light, Intermediate and Tan skin reaching 79–100% at the BED was reported [177]. Lesions in melanocytes may explain the higher risk of lighter skin types of developing melanoma. In addition, they may explain the high prevalence of pigmentation disorders in Tan skin types in individuals of Asian, Hispanic/Latin American and African descent [178,179,180,181,182,183]. 

Although many in vivo and in vitro studies in human and animal models found no difference in DNA repair capacity in skin of different constitutive pigmentation [96,184,185,186,187,188,189], one study [174] showed a greater loss of CPD in skin type IV compared to type II after exposure to sub-erythemal doses suggesting faster repair in skin type IV. In addition, there is now evidence that MC1R can contribute to a protective effect through activation of DNA repair [190,191,192].

### 2.3. Pigmentation Induced by Sun Exposure

**Figure 4:**
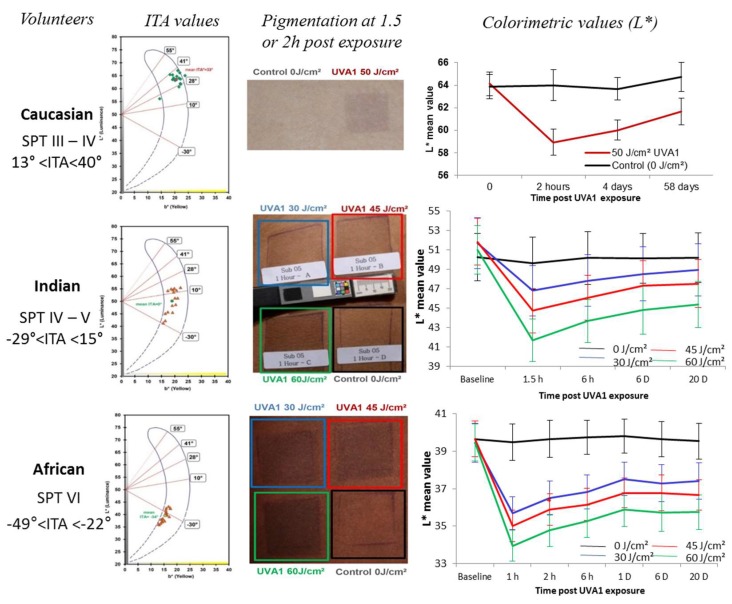
UVA1-induced pigmentation in volunteers with different skin color type. Three groups of volunteers with European (*n* = 24), Indian (*n* = 16) or African (*n* = 22) origin were included. Each volunteer was characterized by its Fitzpatrick phototype (SPT) and ITA value calculated using the colorimetric parameters L* and b* according to the formula ITA° = [ArcTangent((L*− 50)/b*)]180/π. After a UVA1 exposure, pigmentation was visually followed and scored, and measured using colorimetric parameters comparing UVA1-exposed site to non-exposed site. The UVA1-induced pigmentation with a long lasting effect could be observed in the three groups whatever the constitutive pigmentation (adapted from [208]).

The tanning response of human skin to sun exposure can be categorized into three different phases: immediate pigment darkening (IPD), persistent pigmentation (PPD) and delayed tanning (DT). IPD is a reversible transient grayish coloration that occurs during and after UV exposure and fades within minutes to a maximum of 2 h. When the skin is exposed to a sufficient UV dose, IPD is more intense and is followed by PPD that may last 24 h or longer and may blend with the DT response which requires melanin neo-synthesis and occurs between 3–5 days after sunlight exposure [193,194,195,196]. IPD typically appears gray to black while PPD is brown. Both IPD and PPD do not require any new pigment synthesis and are thought to result from oxidation and/or polymerization of pre-existing melanin or melanogenic precursors and metabolites [197,198,199,200]. Melanosomes have also been shown to redistribute within both keratinocytes and melanocytes during the IPD/PPD response [201,202]. PPD is not protective against neither UVB-induced erythema [203] nor UVB-induced DNA lesions [204,205]. For some authors, the IPD/PPD response can develop in all skin phototypes [206,207,208] (Figure 4).

In volunteers from three different ethnic origins (European, Indian, African) and ITA values ranging from 13° to −50°, exposure to 45–50 J/cm² UVA1 induced a similar ΔITA around 16–18° irrespective of the original skin color type [208]. For other authors, there is a lack of IPD in skin phototypes I and II [209]. This may be due to difficulties in evaluating color change in fair-skinned individuals compared to easier observation in higher skin phototypes [206,207,209]. In very fair skinned individuals, this may also be due to the irradiation dose needed to produce IPD/PPD being greater than the dose required to induce sunburn. A lack of IPD response in fair skinned individuals was suggested to be correlated to “non-brown” eye color rather than light hair color [148]. Even if some slight discrepancies could be found between the articles, IPD/PPD action spectrum studies have shown that, in the UV range, the UVA wavelengths are the most efficient with a peak between 340 and 370 nm [193,199]. However, when the source of exposure extends up to 620 nm, short wavelengths of visible light (400–500 nm) appear to be also highly effective [210]. Upon UVA exposure eumelanin undergoes a reversible oxidation of 5,6-dihydroxyindole (DHI and DHICA) moiety to 5,6-indolequinone moiety (for review, see [211]). IPD may fade away through reduction of indolequinone to the original dihydroxyindole moiety. PPD develops by a more complex, irreversible chemical process involving oxidative cleavage of indolequinone to form free pyrrole-2,3,5-tricarboxylic acid (PTCA) [212] and cross-linking of dihydroxyindole that gives pyrrole-2,3,4,5-tetracarboxylic acid (PTeCA) [213]. Both oxidative modifications of eumelanin and cross-linking occur in tandem and lead to increased absorption in the visible range. Pheomelanin also undergoes photodegradation during PPD as UVA exposure induces oxidative conversion of benzothiazine (4-AHP) to benzothiazole (TTCA) moiety. However, in terms of skin pigmentation, pheomelanin may not contribute much to PPD due to its lower content compared to eumelanin [78].

DT is a brown dark yellow pigmentation that appears several days after UV exposure and reaches a maximum after 2–3 weeks depending on the UV dose and skin type [214,215]. It is maximally stimulated by the sunburn/erythema spectrum (290–320 nm) and to a lesser extent by UVA exposure. It results from neo-melanization (de novo melanogenesis) due, to increased activity and number of melanocytes, dendrite branching, tyrosinase activity and number and transfer of melanosomes. Multiple photobiological events initiate melanin synthesis, such as DNA damage/DNA repair [216] membrane damage and factors secreted by surrounding keratinocytes. Activation of P53 protein in keratinocytes as a result of UV-induced DNA insult leads to upregulation of proopiomelanocortin (POMC) which is processed into αMSH. However, the direct regulation of POMC by P53 is still controversial since no P53 responsive element has been identified in the POMC promoter region [217]. Keratinocyte derived αMSH then binds to melanocyte membrane through MC1R resulting in activation of melanogenesis and increased melanin production. Pigmentation resulting from the stimulation of the cutaneous hypothalamic-pituitary axis (HPA) has been studied with regard to the contribution of the different UV wavelengths [218]. UVB was highly effective in stimulation of CRH (Corticotrophin Releasing Hormone), POMC, MC1R, MC2R and β-endorphin whereas UVA was able to stimulate β-endorphin and to a lesser extend POMC. Other keratinocyte derived factors, such as cytokines play a role similar to endothelin 1 (ET-1) or interleukin 1. Membrane effects upon UV exposure are also contributing through release of diacylglycerol (DAG) and subsequent activation of protein kinase C (PKC), a major protein kinase involved in tyrosinase activation. Arachidonic acid metabolites such as leukotrienes C4 or D4 are also known to be mitogenic for melanocytes. 

DT due to resulting increased melanin content has been shown to be slightly photoprotective leading to SPF around 2 [219], based on its protective value from erythema and DNA damage. DT response differs depending on skin color type, as clearly evidenced by the Fitzpatrick classification, at least for skin types I–IV, taking into account the individual tanning ability from “never tan” to “always tan”. Facultative skin color has been shown to be proportional to constitutive skin color [70]. From a mechanistic point of view some articles have addressed the question of differences between skin color types or ethnic origin of the skin. From a global point of view, tanning response whatever the skin color type does not lead to a huge increase in melanin amount. In Caucasians, Asians and Africans, after exposure to 1 MED, the melanin content was not statistically increased one week later, but mostly redistributed in the epidermal layers [220]. The authors suggest that this phenomenon highly contributes to the visible induced pigmentation. In another study, even after three weeks of repetitive UV exposure of subjects phototypes II/III, melanin content increased only slightly without statistically significant levels despite the fact that visible tanning of the skin had increased fivefold, as measured by a chromameter [221]. Alaluf et al. [10,222] confirmed this moderate increase in melanin content in repetitively UV-radiated skin compared to protected skin. In Asian skin, UV tanning response was also reported to be associated with a small increase in pigmentation [94]. Melanocyte density was found not to be increased at seven days after a single 1 MED exposure [220], while after repetitive exposures it has been shown to reach a threefold increase at five weeks [223]. Although pheomelanin synthesis within the melanosome seems to occur prior to eumelanin [224], the amount of melanin observed after UV exposure, appears to result from a slight increase in both eumelanin and pheomelanin in a constant proportion [94,96]. 

Visible light (VL) represents almost half of the solar spectrum and its role in IPD/PPD response has been shown since a long time with a contribution of shorter VL wavelengths around 420–460 nm as shown in IPD/PPD action spectra studies [193,210,225]. Using a solar simulator delivering wavelengths from 385 to 690 nm, the pigmentary doses were established at 80–120 J/cm^2^ in skin phototypes II-IV [226]. More recently, the effects of VL using more controlled VL sources has been revisited. A study compared UVA1 and VL in pigmentation induction [227]. Using a halogen VL source with a higher intensity compared to the sun, the minimal VL pigmentary dose was found at 40 J/cm² and dark skin individuals (SPT IV–VI) responded to VL while no pigmentation was induced in lighter skin type subjects (SPT II). The pigmentation was browner compared to UVA1-induced pigmentation (more greyish) at early time points after exposure and VL was responsible for a more sustained effect. In natural sunlight exposure conditions (Bangalore, India, noon time), the pigmentary effect of VL has been assessed after blocking the UV wavelengths using physical filters. Twenty-five minutes of exposure were sufficient to induce a measurable pigmentation in subjects with phototypes IV-V. In this study, the authors indicate that the contribution of UV and VL seems comparable but with a 25 times higher efficacy of UV (per J/cm²) [228]. To address the respective role of the different VL color subdomains, Duteil et al. used Light Emitting Diodes (LED) with a peak within the shorter wavelengths corresponding to blue-violet light (LEDs emitting around 415 nm) and others with a peak at 630 nm corresponding to red light. They demonstrated that induced-hyperpigmentation in melanocompetent subjects (SPT III–IV) was only observed under blue light exposure with a change in ITA values up to 20 ITA units [229]. They also showed that the mechanisms underlying this pigmentation were different from the UVB-induced pigmentation without contribution of P53 or oxidative stress. To better understand the contribution of VL and UVA1 in induced pigmentation, 10 subjects SPT IV-VI were exposed to pure VL or VL with 0.5% UVA1 and assessments were performed from 24 h to 14 days post exposure. Pigmentation induced by both types of exposure indicate that, when combining the two wavelength domains an additive effect could be observed with regard to pigmentation intensity but erythema was only seen where skin was exposed to VL + UVA1 [230]. The mechanisms underlying blue light-induced pigmentation has been recently reported to involve the presence in the melanocyte membrane of a light photoreceptor belonging to Opsins family, the OPN3 well known to detect blue light through the retina [231]. Activation of OPN3 by blue light stimulates the calcium flux within the melanocyte which in turns activates MAP kinase pathways leading to increased melanogenesis. Interestingly, OPN3 activation was also responsible for the formation of TYR complexes with other melanogenic proteins, leading to a sustainable Tyrosinase activity, and this specifically in dark skin melanocytes (SPT III–VI) compared to lighter ones (SPT I and II). 

The role of solar exposure is considered in the dermatological practice to be an important contributor to pigmentary disorders and efficient photoprotection is usually recommended. The development of protocols aiming at testing the efficacy of VL blocking agents has also been recently published [232]. 

## 3. Mid- and Long-Term Consequences of UV Exposure

### 3.1. Skin Cancer

UVR exposure causes skin cancer including carcinomas, i.e., basal cell carcinoma (BCC) and squamous cell carcinoma (SCC) which are keratinocyte-derived cancers and, more dramatically, malignant melanoma (MM), which arise from melanocytes, and are responsible for the vast majority of skin cancer deaths due to metastasis proneness (American Cancer Society, 2016). Epidemiologic studies show higher incidences of BCC and SCC as well as MM in Caucasians compared to African-Americans [233,234,235,236,237]. Recent data from South African population demonstrated that populations of African and Asian ancestry had a lower incidence of SCC, BCC and MM than people of mixed European descent and considerably lower than individuals of European descent [176]. A 60-fold and 20-fold higher incidence of BCC and SCC, respectively, was reported in individuals of European ancestry compared to African ancestry. In the United States, the rates of melanoma have been shown to vary with ethnicity and, between 2010 and 2014, the age-adjusted incidence rates per 100,000 in men and women were 34.4 and 20.9, respectively, in white individuals; 5.0 and 4.7, respectively, in Hispanics; 4.3 and 4.9, respectively, in American Indian/Alaskan natives; 1.6 and 1.2, respectively, in Asian/Pacific Islanders; and 1.1 and 1.0, respectively, in black individuals [238].

Although the risk of skin cancer is lower in darker skin compared to lighter skin types [239], UVR exposure is a risk factor for skin cancer even in darker skin types including African Americans, Asians and Hispanics [235,240,241,242,243] and epidemiologic studies have shown that sunburn occurs even in darkly pigmented skin [188,197].

The lower rates of skin cancers found in pigmented skin can be explained by photoprotection provided by melanin. Dark skin has been shown to have an intrinsic UVB protection factor of 13.4, and a UVA protective factor of 5.7 compared to 3.4 and 1.8, respectively, in light skin. Additionally, dark skin transmits 7.4% of UVB and 17.5% of UVA, compared to 29.4% and 55.5%, respectively, in light skin [158,159,160]. 

Even though skin cancer is less frequent in nonwhite/dark skinned individuals, it is often associated with increased morbidity and mortality due to the fact that some perceive themselves to be at a lower risk for developing sunburn and skin cancers [244,245]. There also is a lack of awareness of these diseases among these populations, associated with delayed diagnosis [240,246,247,248,249,250,251,252]. 

### 3.2. Photoaging

**Figure 5:**
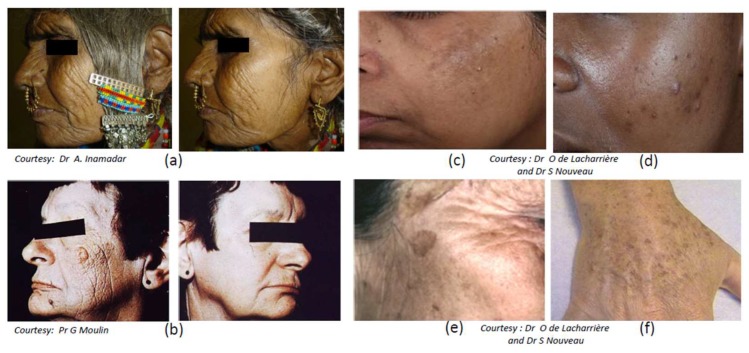
Photoaging and pigmentary disorders in skin of various phenotypes. Photoaging: (**a**) in Indian and (**b**) Caucasian (North European) women. Four types of major hyperpigmented disorders linked to sun exposure: (**c**) melasma; (**d**) post-inflammatory hyperpigmentation (PIH) (acne marks); (**e**) seborrheic keratosis; and (**f**) solar lentigo.

Skin aging involves two types of processes, intrinsic aging (chronological aging) and extrinsic aging also called photoaging, considered as a premature skin aging and resulting from the impact of environmental stresses, essentially solar UV exposure. The overall aging signs have been shown to differ depending on the ethnic origin and related to facial structural differences (for reviews, see [253,254]). In this section a focus on photoaging is made. Although clinical manifestations of skin photoaging include pigmentary disorders, those aspects are detailed in a specific section. One of the major clinical traits of photoaging is the progressive appearance of coarse wrinkles associated with the formation of dermal solar elastosis, (Figure 5a,b), which results from alterations and reorganization of the whole dermal structure. 

The entire UV spectrum is involved in photoaging. UVA wavelengths have been shown to highly contribute due to their high penetration properties, which allows them to directly reach the dermal compartment. Most of the literature on this subject is related to ethnic origin rather than to properly classified skin color phototypes. More than differences in the nature of clinical signs, photoaging differences can be found regarding the age of onset and the severity of damage that can be explained by the fact that UV and especially UVB rays penetrate in different amounts depending on skin color type and melanin content. Wrinkling process appears to highly depend on skin color type being delayed in darker skin types (African Americans) compared to Caucasians [255]. In addition to lower amounts of UV reaching the dermis, darker skin types have been shown to have a thicker dermis, which has more elasticity and resistance [254]. Clinical manifestations of aging appear 10–20 years later in pigmented skin populations compared to age-matched white populations [158]. A 10 year delay has also been found between Chinese and European populations [178]. In Asians, although the principal clinical signs of skin phototoaging are pigmentary changes, wrinkle patterns can also develop [256,257] even if due to differences in sun exposure habits it is not possible to strictly compare European and Asians photoaging process. Wrinkle patterns in Asians are characterized by coarser, thicker and deeper wrinkles compared to Caucasians [258]. Wrinkling in Asians women is reported as not noticeable before the age of 50 and that the degree is less severe compared to European women [259]. However, in both Caucasian and Asian populations the biological events related to the photoaging process seem quite similar with an upregulation of matrix metalloproteinases (MMPs), especially MMP1 and decreased collagen content resulting from down-regulation of expression of main structural collagens notably Col1 and Col3 [260,261]. Although the mechanisms seem quite similar, one study showed a higher MMP1 increase in lighter skin phototypes (I/II) compared to darker skin types (V/VI) after UV exposure [262]. Another study found that the induction of MMP-1 and MMP-9 to erythemal doses of SSR is similar in all skin types from I to VI but different when the same physical doses were delivered with increase in MMPs in lighter skin types and not in skin type VI [169]. 

### 3.3. Skin Pigmentary Disorders Linked to Sun Exposure

Beyond the tanning effect induced by acute sun exposure, pigmentary disorders can be triggered or exacerbated by mid or long-term sun exposure. Pigmentary disorders, even benign, cause psychosocial distress and negative impact on self-image and the quality of life of individuals [263]. Type, onset, frequency and severity of hyperpigmented lesions depend on the skin complexion and on individual ancestry. High prevalence of pigmentary disorders in dark skinned people (South/southeast Asian, Hispanic/Latin American, African descent) has been reported [179,181,182,183,264]. Pigmentary disorders are the third and fourth more common dermatoses in Blacks and Hispanics respectively [181]. However the special importance of skin hyperpigmentations in Asian populations, from lighter to darker skin tone has also been however highlighted [178,259,265,266,267]. Hyperpigmented spots develop earlier and/or are more pronounced in Asian than in Caucasian population. A higher spot prevalence was reported in Chinese women than French women, both of them having mostly intermediate skin complexion [178]. In addition, over the age of 40, Chinese women who have pigmented spots, have many spots, whereas, in France, women have only a few spots. These results indicate that pigmented spot intensity is an earlier ageing feature in Chinese women than in French women. 

A focus is made on four major hyperpigmented disorders linked to the light exposure: melasma, post-inflammatory hyperpigmentation (PIH), seborrheic keratosis (SK) and solar lentigo (SL) (Figure 5c–f).

#### 3.3.1. Melasma

Melasma is a chronic acquired hypermelanosis characterized by tan, dark or grayish- brown macules and patches with bilateral disposition and irregular boarders in sun-exposed areas, especially the face. The most common facial location are the cheeks, forehead and upper lip. 

Epidemiological studies have reported higher prevalence among pigmented phenotypes, mainly in SPT III–V, but rarely in extreme skin type VI [182,183,268,269]. The more affected populations are East Asians (Japanese, Korean and Chinese), Indian, Pakistani, Middle Eastern and Mediterranean-African, Hispanic-Americans and Brazilians. In India, 20–30% of 40–65-year-old women have a facial melasma [77]. Family history, particularly in darker skin, is an important risk factor in the development of melasma emphasizing the influence of the genetic background: in a worldwide survey on 324 women, about 50% of individuals with melasma, have at least one family member with the pathology [270]. 

Formerly, melasma was classified by Wood’s lamp examination as epidermal, dermal or mixed type according to the pigment location. However, from histology and reflectance confocal microscopy analysis, increased pigmentation throughout the lesional epidermis was found as a hallmark of melasma irrespective of the skin type and the ethnic origin [271,272,273,274]. Upregulation of melanogenesis related genes (TRP1, TRP2, and MITF) and histological observation of hypertrophic melanocyte attest the activation of epidermal melanocytes [273,274]. The presence of melanin in the dermis seems to be also dependent on the skin type with no detection in Caucasian [274] compared to common and significant amount in melasma patients with SPT III–V [271,272,275]. However, dermal melanin can also be visible in non-lesional high SPT skin [272]. In parallel, in darker skin, melanophages were detected in the superficial dermis with a very heterogeneous distribution varying from one melasma region to another for a same patient and even inside a given melasma [273]. 

Along with genetic predisposition, melasma etiology has been associated with female sex hormones for long time, since melanogenesis may be stimulated by a high level of estrogen and progesterone [270]. The main differentiating factor in terms of hormonal status at the onset of melasma is skin type with a constant frequency of melasma appearance during pregnancy in all skin types but a higher prevalence of the onset of melasma post-pregnancy in skin types III–IV [270]. However, since melasma may also affect men, with family and sun exposure histories, female sex hormones might not be an essential causal factor of the disease [276]. In contrast, acute sun exposure is the most important triggering environmental factor of melasma with a constant worsening of the disorder during the summer season. Additionally, high incidence is found in populations living in intertropical areas or in elevated altitudes where there is greater exposure to UVR [182,183,268]. The involvement of UVB and UVA is demonstrated by the reduction of both the intensity and the incidence of the disease with the use of a broad spectrum sunscreen [277]. Visible light (blue-violet light) also contributes to the lesion since the addition of iron oxide (blue light blocking agents) to a broad UVB-UVA sunscreen prevented the relapse [278]. Furthermore, recent knowledge on chronic impact of sun exposure on the pathogenesis of melasma, suggests that melasma displays hallmarks of photoaging. Histological analysis showed increased solar elastosis, basement membrane disruption, rete ridge flattening, increased microvasculature, infiltration of mast cells and subclinical inflammation [279]. Transcriptomic analysis revealed dysregulation of numerous genes and especially some actors of the Wnt pathway produced by fibroblasts [274]. Altogether, these data suggest that melasma is not only a melanocyte disorder but that other actors such as the dermal compartment might also play a role in its physiopathology and that long-term exposure to sun radiation might also be a key process. Indeed, patients with fair skin phototypes tend to develop melasma earlier in life [270]. This is explainable by the natural photoprotective role of melanin which may delay the onset of melasma in darker skin types. Reviewing epidemiological and pathophysiological data, Passeron and Picardo [280] proposed the new paradigm of melasma as being a photoaging skin disorder affecting genetically predisposed individuals. 

#### 3.3.2. Post-Inflammatory Hyperpigmentation (PIH)

Post-inflammatory hyperpigmentation is induced by various inflammatory processes which can be endogenous or exogenous. Endogenous causes of PIH include atopic dermatitis, acne vulgaris, psoriasis, or pseudo-folliculitis barbae (razor bumps) and exogenous causes include insect bites, laser procedures, chemical peels or drug sensitization. PIH appears as macules or patches commonly observed on sun exposed body areas: face (cheeks, mandibular area, forehead, temples), back and front shoulders/trunk. It has been recently proposed to classify PIH into two groups: epidermal or dermal according the melanin pigment deposition [281]. The characteristic response of melanocytes to inflammatory stimuli is hyperplasia and hyperactivity of melanocytes, via the generation of inflammatory mediators (eicosanoids, cytokines, ROS, endothelin-1, stem cell factors), but without increase of their number. Infiltrates of lymphohistiocytes and melanophages at proximity of the blood vessels and in the dermal papillae, as well as upregulation of MMP2, a basement membrane collagen IV degradative enzyme, are observed in the two types, however more intensively in the dermal PIH. Infiltration of mast cells could be observed only in the dermal PIH subset [281]. Epidermal PIH induces dark brown lesions while dermal PIH causes brown to blue/gray discoloration of the skin. 

PIH affects all skin types but numerous studies reported that PIH is more common in pigmented skin and among pigmentation disorders in African, Asian and south American ancestry, PIH is the most common diagnosis [182,264,265,282,283,284,285]. Intensity and frequency of the hypermelanosis are higher in individuals with dark skin complexion due to the increased reactivity of melanocytes. In Asia, Malaysians and Indians (living in Singapore) have a higher incidence of PIH than lighter skinned Chinese individuals showing the importance of the degree of constitutive pigmentation in the development of PIH [265]. Pigmentation problem is often long lasting and may be permanent (for dermal PIH). More than half of the subjects reported PIH lasting for one year or longer, and more than 20% up to five years [286]. 

PIH is a common sequela in acne patients that persists for months after the acne lesion itself has resolved [287]. Non-Caucasian women declared to be more bothersome by the PIH than by the acne itself. Prevalence of PIH in individuals with acne was reported to be 65% for African-American, 52.7 for Hispanic and 47.4% for Asian [14]. In a more recent study performed in seven countries, from north to south Asia including mostly SPT III–IV individuals, around 60% of patients consulting for acne had PIH [286]. 

PIH complications frequently occur after procedural therapies (laser, IPD, peeling) with a considerably higher risk in people of color [288]. PIH prevalence was around 20% in Asians treated for solar lentigines using Q-switched laser [289,290] and 11–17% after laser resurfacing [291]. Compared to the 23% of SPT I–III patients who developed PIH after ablative fractional carbon dioxide laser treatment, the incidence of PIH was much higher in phototype IV patients since it reached up to 92% [292,293]. Chemical peels and microneedling should be also used with caution in people of color due to the higher PIH induction risk [294,295]. 

In any case, sun exposure is a key factor by worsening or triggering the development of PIH. PIH occurs on photoexposed skin and UV and visible light (blue light) are known to stimulate pigmentation, even more in the darker individuals (see Section 2.3*).* In a clinical PIH model (suction blister) hyperpigmentation only occurred when skin was maintained exposed to ambient light [296]. 

Extremely frequent, more intense and taking months or years to resolve, PIH represents one of the most common dermatological complaints among darker skinned individuals [282,285,297]. 

#### 3.3.3. Seborrheic Keratosis (SK)

Seborrheic keratosis is the most common benign skin tumor of middle-aged and elderly adults, affecting nearly 83 million individuals in the US alone. Most individuals with SKs have multiple lesions [298]. They clinically appear as acquired well demarcated, flat (macular) or elevated/verrucous patches or plaques, stuck on the skin, varying in color from yellow to black. Whether flat forms are initial SKs which grow thicker over time and become verrucous is not defined. The main clinical variants of seborrheic keratosis are: common seborrheic keratoss (CSK) dermatosis papulosa nigra (DPN), flat SK, pedunculated SK and stucco keratosis [299]. Thorough dermatoscopic criteria have been defined to non-invasively classify SKs and distinguish them from malignant lesions [300]. Histopathologically, SKs are classified into six types: acanthotic, hyperkeratotic (verrucous), adenoid, irritated, clonal and reticulated. Different subtypes are frequently combined in the same lesion. Proliferation of basaloid cells leads to acanthosis which along with papillomatosis, is the main characteristic of the disorder which shows also varying extents of hyperkeratosis, pigmentation, horn or pseudo-cysts and inflammatory cell infiltrates. One third of SK may be hyperpigmented. Despite being hyperproliferative, SKs respect the basement membrane, remain differentiated and do not evolve into malignancy. Due to the broad clinical and histological heterogeneity of SKs as well as the nonsystematic description of the studied subtypes, elucidation of the pathogenesis and risks factors remain highly difficult. Nevertheless, SKs seem to be linked to the clonal expansion of somatically mutated cells. These are genetically stable despite harboring one or multiple alterations of various oncogenes as example *FGFR3, PIK3CA, CDKN2A, TERT, HRAS, KRAS, EGFR, AKT1* [301,302]. 

Although SK frequency is very high, little is known about the epidemiology of the lesion. A possibility of genetic predisposition has been raised but clear demonstration is still lacking. Patients with a large number of lesions often have a family history of SK [298]. Lighter skin seems to be more prone than darker skin to SKs where they are rare, except for the Dermatosis Papulosa Nigra (DPN) subtype which is very common among people of color [303]. Considered as a variant of SK in people with dark skin, DPN also manifests at earlier ages than CSK, is more common among women and presents as multiple heavily pigmented papules over the face, neck, chest, and upper back [299,304]. 

SKs are shown to be associated with increasing age [299,305,306,307]. More than 90% of people over 50 years was found to have SK in Korea and up to 100% in Australia [305]. Despite the positive correlation between age and prevalence, SK can also appear at relatively young ages [305,308]. In Great Britain, 17% of women under the age of 40 had at least one SK. 

SKs affect more commonly the sun exposed areas (head near the temples, neck, chest, back, hands, forearms). On photoexposed skin, SKs frequency and size are higher than on non-exposed areas and they are more often flat [305,306,308]. Besides the high frequency, the earlier SK onset on photoexposed sites reinforces the fact that sun exposure may be an exogenous risk factor. Under the age of 40 years, more Australian than English people had SKs and in Australia, up to 24% of subjects between 15 and 30 years old have at least one SK. Actually, the mutational pattern depicted typical UV signature with majority of C>T and CC>>TT base changes at dipyrimidic sites which concurs with self-reported history of sun exposure at the lesion site [302]. Lifetime cumulative sun exposure of more than 6 h per day has been associated with a 2.3-fold higher risk of SKs than a sun exposure of less than 3 h per day [306]. This underlies the high frequency of SKs in younger people in Australia where the sunlight exposure is high [308]. Altogether, these findings show that aging and cumulative sun exposure are independent contributory factors to the development of SK. 

#### 3.3.4. Solar Lentigo 

Solar lentigo (also called age spots, actinic lentigo or senile lentigo) is a brownish pigmented macule with irregular borders and a size of few millimeters. It is frequently found on photoexposed areas such as the hand, face, and upper back. Histological observations confirmed that melanin accumulates in the actinic lentigo epidermis mostly in the basal layer [309,310,311]. Such lentigo is also characterized by deep epidermal invaginations that form club-shaped or bud-like extensions into the dermis. Melanocyte activation is controversial, as their density along the dermal epidermal junction has been found to be similar in lesional compared to perilesional skin [310,311]. Of interest, transcriptomic studies revealed multiple molecular alterations notably dysregulation of keratinocyte proliferation and differentiation and modifications of the dermal extracellular matrix suggesting that keratinocytes and the dermal compartment could play a crucial role in the physiopathology of lentigines [310,311,312].

Lentigines have been clearly associated with age and sun exposure [313,314,315,316] and represent a hallmark of photoaged skin. Lentigines on the face was significantly associated with cutaneous signs of photodamage, i.e., elastosis. Depending on the body area, specific differences in the etiology of lentigines seem to occur. Lentigines on the face or hand could result from cumulative sun exposure and not from sunburn events. Solar lentigines on the back have been associated with frequent sunburn and with recreational sun exposure but not with life time exposure. Onset of pigmented spots can nevertheless be early: in Indian women, after the age of 30, lentigines were present in more than 80% of women [77].

Phototypes II–IV are determined as risk factor for lentigines. Monestier et al. [317] showed that the presence of multiple senile lentigos on the face, a skin ageing feature, was associated with SPT III and IV. More recently, lentigines were identified to be associated with SPT II and III [315]. In this study, lentigines were also significantly more frequent in women who declare to achieve a dark/very dark sun-tanning and had a history of facial freckles. 

To investigate genetic predisposition for lentigines, studies on Japanese and German women JAGE cohort (39 German, 48 Japanese; mean age of 49 years) and on 372 German women SALIA cohort (mean age of 74 years) were first conducted [267]. The results confirmed the higher prevalence of lentigines occurred in Asian compared to European populations [178,318]. Japanese women showed 4.3-fold more lentigines on cheeks but fewer lentigines on the arms than age-matched German women. No difference was observed on the forehead. Genotyping 25 single-nucleotide polymorphisms relevant to melanin synthesis in eight genes (*MCR1, ASIP, TYR, TRP1, TRP2, TPCN2, P, SLA45A2*), identified a correlation between one genetic variant (rs26722), and for the allele A, in the *SLC24A5* gene and the occurrence of lentigines. More recently, a GWAS study performed on facial pigmented spots including mainly lentigines (and a minority of SK), in two cohorts of North Europeans (2844 individuals) concluded that genetic variations in 4 genes *IRF4, MC1R, RALY/ASIP*, and *BNC2* could contribute to the acquired amount of facial spots during aging [319]. Strikingly, skin color-adjusted analyses showed that these gene variant associations were independent of the impact of the genes on skin color pathways. The association between the *IRF4* gene and the severity of solar lentigines on cheek or forehead of 502 middle aged French women was replicated [316] but pointed out novel genetic signals in the *MITF* and *HLA-C* genes for solar lentigines on the forehead, however the findings remain controversial [320]. Insights on genetic predisposition to develop pigmented spots are emerging and may be helpful to unravel the molecular mechanisms underlying their development. Robustness and relevance of the data will gain by their confirmation in increased-sized cohorts and by accurate description/characterization of the studied spot types (solar lentigines and SK are easily confused).

#### 3.3.5. Management of Hyperpigmented Disorders Linked to Sun Light

The first line of treatment is to avoid the causative or aggravating factor, i.e., sun light exposure, by using sun blocking agents. Such protection has shown to reduce the incidence or relapse of melasma [277,278]. Even though the use of sunscreen of SPF 30 or 60 in African-American and Hispanic individuals resulted in a decrease of preexisting hyperpigmentation [321], only 50% of patients with PIH use sunscreens [263]. Thus, the use of broad spectrum sunscreen needs to be encouraged in all types of skin, even in darker skin, for prevention and for sustaining complementary therapies.

In association with UV blocking agents, the second line of treatment may be physical therapies (cryotherapy, laser, IPD, dermoabrasion) or chemical peels which are quite efficient for the treatment of hyperpigmentation [322,323]. However, they are usually not sustainable and associated with relapses. The risks of side effects such as hypo- or hyperpigmentation (PIH) specially in dark skins phototypes is also high with these procedures. Alternatively, less invasive and costly than physical modalities, topical or oral treatments , combining depigmenting agents with different modes of action may be useful [322,323,324]. They may contain different molecules acting at different level of melanogenesis: (1) inhibitors of tyrosinase (e.g., hydroquinone, mequinol, rucinol, arbutin, kojic acid, azelaic acid or natural extracts such as pomegranate extract rich in ellagic acid); (2) inhibitors of tyrosinase maturation (e.g., N-acetyl glucosamine); (3) inhibitors of melanosome transfer (e.g., niacinamide, soybeans, lectins). Other agents can be used to complete the action of melanogenesis such as agents accelerating epidermal desquamation and melanin turn over (e.g., retinoids, salicylic acid, hydroxy acids) or agents acting by other modes such as anti-inflammatory and/or anti-vasculature agents (e.g., dexamethasone, fluocinolone acetate, and tranexamic acid) or antioxidants (e.g., vitamin C, resveratrol, vitamin E, ferulic acid, natural agents such as grapeseed extract, and gingko biloba). 

Most of the depigmenting topical treatments use an inhibitor of tyrosinase, the rate limiting enzyme of melanogenesis. Hydroquinone is the gold standard however with safety issues. It is noteworthy that a new potent inhibitor of human tyrosinase showing efficiency on age spot has been recently developed [325]. 

Combined therapies are widely used. Di or tri-combined therapy using hydroquinone, tretinoin or a topical corticosteroid (fluocinolone acetate or dexamethasone) showed good efficacy for medical treatment of melasma, PIH or solar lentigines with some limiting aspects (long-term treatment, side effects or relapse) [323,326,327,328,329]. Interestingly, a new and safe whitening formula containing ferulic acid, Ginkgo Biloba, lipohydroxyacid, niacinamide and thermal spring water significantly improved melasma after a three-month-treatment period compared with placebo [330].

Besides, the development of natural extracts and/or devices aiming at increasing the delivery of actives into the skin is a growing trend. 

To sum up, hyperpigmented disorders contribute to an overall unevenness of skin pigmentation, in particular in areas exposed to sunlight. Even if not sufficient, advances in the etiology, clinical and biological characterization evidence their broad variety and their specificity according to skin complexion. Globally, robustness and relevance of the data will gain with large-sized cohort studies and with accurate clinical description and selection of the lesions (lentigines and SK are easily confused). To increase the efficacy of existing treatments or overcome their lack of long lasting efficacy leading to relapse, tailored treatments of hyperpigmented disorders according to the skin complexion and origin is the new challenge. 

## 4. Conclusions

This review aims at describing the known key factors involved in skin pigmentation diversity and the related response to solar exposure. Although growing knowledge is available particularly with the contribution of new genotyping and imaging technologies, there are still gaps requesting further studies to optimize the reliability of skin color classification or to decrypt pigmentary disorders physiopathology. In parallel, world globalization raises crucial and new questions concerning health and beauty issues as a consequence of acute or daily chronic sun exposure. While fair skins were in the past considered as the only sun sensitive phototypes due to higher skin cancer prevalence, nowadays, concerns regarding pigmentary problems of people with darker skin are emerging. The complexity of the subject results from the combination of various sun exposure conditions and skin color type diversity and needs further research in the field of personalized photoprotection for adapted recommendations. The development of personal UV dosimeters as well as the ability for everybody to better characterize its own skin color type are key future issues. The better understanding of the contribution of the different solar wavelengths and their biological effects will also improve the prevention and correction of the clinical impacts through the development of dedicated products with higher performance. 

## Figures and Tables

**Table 1:** SNP positive selection per gene and population. Gene locus, SNP (ancestral > derived allele), population and reference are indicated.

Gene	SNP	Population	Reference
MC1R	rs1805005 G>T	Europeans	[33]
	rs1805006 C>A	Europeans	[33]
	rs1805007 C>T	Europeans	[33,34]
	rs1805008 C>T	Europeans	[33,34]
	rs1805009 G>C	Europeans	[33]
	rs2228479 G>A	Europeans	[33]
	rs11547464 G>A	Europeans	[33]
	rs1110400 T>C	Europeans	[33]
	rs885479 G>A	Europeans	[33]
SLC24A5	rs1426654 G	Africans, Melanesians	[35,36]
		East Asians, Southeast Asians	[35,36]
		Americans	[35,36]
	rs1426654 G>A	Europeans	[33,35,36]
		South and Central Asians, Middle East, Pakistani, Iran, North India	[33,35,36]
		North Africa, Ethiopia, Tanzania, Botswana	[37]
	rs1426654	Eurasians	[38]
	rs2470102	KoeSan (Southern Africa)	[38]
SLC45A2	rs16891982 C	Africans, Melanesians	[35]
		East Asians, Southeast Asians	[35]
		Americans	[35]
	rs16891982 C>G	Europeans	[33,35]
		Middle East, Pakistani	[35]
		North Africa,	[35]
	rs26722 G>A	Asians	[33]
OCA2	rs1800407 G>A	Europeans	[33]
	rs1667394 A	Europeans	[34]
	rs7495174 A	Europeans	[34]
	rs1800414 A>G	East Asians	[33,39,40]
	rs1800404 C	Africans, sub-Saharian Africans	[33,37]
		South Asians, East Asians	[33,37]
		Australo-Melanesians	[33,37]
	rs1800404 C>T	Europeans	[33,37]
		Asians	[33]
		Americans	[33]
		South Africans	[37]
ASIP	rs4911442 A>G	Europeans	[33]
	rs6058017 A>G	Europeans	[33]
	rs1015362 G	Europeans	[34]
	rs1015362 A>G	Africans	[33]
	rs 6058017 G	African Americans	[41]
	rs 6058017 G>A	Europeans	[33]
TYR	rs1042602 C>A	Europeans	[33]
	rs 1800422 G>A	Europeans	[33]
	rs1126809 G>A	Europeans	[33]
	rs1393350 A	Europeans	[34]
TYRP1	rs1408799 C>T	Europeans	[33]
	rs2733832 C>T	Europeans	[33]
	rs34803545	KoeSan (South Africa)	[38]
KITLG	rs642742 A>G	Africans	[33]
	rs12821256 T>C	Europeans	[33,34]
IRF4	rs12203592 C>T	Europeans	[33,42]
	rs12203592 C	Chinese, Japanese	[42]
		Africans	[42]
	rs1540771 G>A	Europeans	[33]
SLC24A4	rs12896399 G	Europeans	[42]
	rs12896399 G>T	Europeans	[33,34]
		Asians	[33]
MFSD12	rs56203814 C>T	Africans, East Africans	[37]
	rs10424065 C>T	Africans, East Africans	[37]
	rs6510760 G	Europeans	[37]
		East Asians	[37]
		Ethiopians, Tanzanians	[37]
	rs6510760 G>A	sub-Saharians Africans	[37]
		South Asians	[37]
		Autralo-Melanesians	[37]
	rs112332856 T	Europeans	[37]
		East Asians	[37]
		Ethiopians, Tanzanians	[37]
	rs112332856 T>C	sub-Saharians Africans	[37]
		South Asians	[37]
		Autralo-Melanesians	[37]
DDB1	rs7120594 T		[37]
	rs11230664 C	East Africans	[37]
		South Asians	[37]
		Australo-Melanesian	[37]
	rs11230664 C>T	Europeans	[37]
		East Asians	[37]
		Native Americans	[37]
TMT38	rs7948623 A	non Africans	[37]
	rs7948623 A>T	East Africans	[37]
		South Asians	[37]
		Australo-Melanesian	[37]
HERC2	rs4932620 C>T	Ethiopians, Tanzanians	[37]
		South Asians	[37]
		Austro-Melanesians	[37]
	rs 6497271 A	South Asians	[37]
		Australo-Melanesians	[37]
		Africans	[37]
	rs 6497271 A>G	Europeans	[37]
		South Africans	[37]
SMARCA2	rs7866411	KoeSan (South Africa)	[38]
VLDLR	rs2093385	KoeSan (South Africa)	[38]
SNX13	rs2110015 >T	KoeSan (South Africa)	[38]

## Abbreviations

2-SCD	2-*S*-cysteinyldopa
4-AHP	4-Amino-3-hydroxyphenylalanine
5-SCD	5-*S*-cysteinyldopa
ACTH	Adrenocorticotropic hormone
ADAM17	ADAM metallopeptidase domain 17
ADAMTS20	ADAM metallopeptidase with thrombospondin type 1 motif 20
AKT1	AKT Serine/Threonine Kinase 1
ASIP	Agouti signaling protein
ATRN	Attractin
BCC	Basal cell carcinoma
BED	Biologically efficient dose
bFGF	Basic FGF
BNC2	Basonuclin 2
CIE	Commission Internationale de l’Eclairage
CPD	Cyclobutane pyrimidine dimer
DCT	DOPA chrome tautomerase
DDB1	Damage specific DNA binding protein 1
DHI	5,6-Dihydroxyindole
DHICA	5,6-Dihydroxyindole-2-carboxylic acid
DNA	Deoxyribonucleic acid
DNA-PF	DNA-Protection factor
DOPA	l-3,4-dihydroxyphenyilalanine
DRS	Diffuse reflectance spectroscopy
DT	Delayed tanning
DTNBP1	Dystrobrevin binding protein 1
ECM	Extracellular matrix
EDA	Ectodysplasin A
EDN3	Endothelin 3
EGFR	Epidermal growth factor receptor
EI	Erythema index
FGFR3	Fibroblast growth factor receptor 3
FOXN1	Forkhead box N1
GM-CSF	Granulocyte-macrophage colony-stimulating factor
GWAS	Genome-wide association study
HERC2	HECT And RLD domain containing E3 ubiquitin protein ligase 2
HGF	Hepatocyte growth factor
HLA-C	Major histocompatibility complex, class I, C
HPLC	High-Performance liquid chromatography
IL1α	Interleukin 1 alpha
IPD	Immediate pigment darkening
IR	Infrared radiation
IRF4	Interferon regulatory Factor 4
ITA	Individual typology angle
KITLG	KIT ligand
LYST	Lysosomal trafficking regulator
MATP	Solute carrier family 45, member 2
MC1R	Melanocortin-1 receptor
MED	Minimal erythemal dose
MFSD12	Major facilitator superfamily domain containing 12
MI	Melanin index
MITF	Microphthalmia-associated transcription factor
MM	Malignant melanoma
MYO5A	Myosin VA
OCA1	Oculocutaneous albinism type 1
OCA2	Oculocutaneous albinism type 2
OPN3	Opsin 3
PIH	Postinflammatory hyperpigmentation
PIK3CA	Phosphatidylinositol-4,5-bisphosphate 3-kinase catalytic subunit alpha
PKA	Protein kinase A
POMC	Proopiomelanocortin
PPD	Persistent pigment darkening
PTCA	Pyrrole-2,3,5-tricarboxylic acid
ROS	Reactive oxygen species
SBC	Sunburn cell
SCC	Squamous cell carcinoma
SK	Seborrheic Keratosis
SLC24A5	Solute carrier family 24, member 5
SLC45A2	Solute carrier family 45, member 2
SMARCA2	SWI/SNF related, matrix associated, actin dependent regulator of chromatin, subfamily A, member 2
SNP	Single nucleotide polymorphism
SNX13	Sorting nexin 13
SPF	Sun protection factor
SPT	Skin phototype
SSR	Solar simulated radiation
TERT	Telomerase reverse transcriptase
TMEM38	Transmembrane protein 38
TPCN2	Two pore segment channel 2
TRP1	Tyrosinase-related protein 1
TRP2	Tyrosinase-related protein 2
TTCA	Thiazole-2,4,5-tricarboxylic acid
TYR	Tyrosinase
UVA	Ultraviolet radiation A
UVB	Ultraviolet radiation B
UVR	Ultraviolet radiation
VLDLR	Very low density lipoprotein receptor
αMSH	Alpha-Melanocyte-stimulating hormone
